# Correspondence

**Published:** 1871-11

**Authors:** 


### Correspondence.

---

Messrs. Editors: Permit me to call attention through the dental journals to the fact that but a few months only of the Goodyear patent now remains unexpired to bother the dentists.

The Goodyear patent expires next May. Its last extension was a crying shame, but nevertheless we had to submit to it. The Cumming's Patent is as worthless as straw, and I trust no dentist will respect it, notwithstanding it has been coupled with the Goodyear patent in an *ex parte* license contract, and its recognition enforced by the Rubber Company upon the dentists. The Cumming's Patent applied for and issued long after the general introduction of rubber into the profession, has no claim whatever upon us, nor are all those licenses which we have been obliged to sign, at all expressive in any sense of any choice on our part, nor have we had anything to say about a mutual agreement, but simply to accept their terms, or be deprived of the use of the rubber. On the principle of a highway robber they have said to us, Your money, or you will be sorry! I think every dentist will regard his contract with the Rubber Company as binding as he would a promise to a highway robber, and I can not think he would respect it much more. A case has occurred where the Vulcanite Company immediately abandoned their cause upon the filing of a replication, in a suit where the Cumming's Patent alone was in question. I refer to the case of Dr. T. B. Gunning, of New York (and I wish that replication, which is in print, might be published in the dental journals). It is a very able defense and covers the whole ground, and gives the Cumming's Patent no foundation, in fact, to stand upon. No doubt a shrewd plan of getting each and every dentist committed for a year or so, will be introduced by the Rubber Company at the commencement of the New Year, although they well know they have but four months or so wherein they can make any valid claim upon us.

Let each decide what he will do before the New Year comes in.

Many can relinquish the use without doubt for that short time. All ought to use freely after the expiration of the time, without any royalty to anybody. It will be wise to have a fund to try this issue with the Rubber Company (if they see fit to press the matter) by a combination of the dentists, which will have every prospect of success if there is any justice to be had.

Respectfully,

One of the Pioneers in Dental Rubber Work.

